# First-night effect on sleep time in dairy cows

**DOI:** 10.1371/journal.pone.0195593

**Published:** 2018-04-13

**Authors:** Emma Ternman, Matti Pastell, Laura Hänninen, Sigrid Agenäs, Per P. Nielsen

**Affiliations:** 1 Department of Animal Science, Aarhus University, Tjele, Denmark; 2 Natural Resources Institute Finland (Luke), Green Technology, Helsinki, Finland; 3 Department of Production Animal Medicine, University of Helsinki, Finland and Research Centre for Animal Welfare, Helsinki, Finland; 4 Department of Animal Nutrition and Management, Swedish University of Agricultural Sciences, Uppsala, Sweden; 5 Department of Veterinary and Animal Sciences, University of Copenhagen, Frederiksberg C, Denmark; University of Illinois, UNITED STATES

## Abstract

In human sleep studies, the probability of discomfort from the electrodes and the change in environment usually results in first-night recordings being discarded. Sleep recordings from the first night in human subjects often differ in amount of REM (rapid eye movement) sleep and the overall sleep architecture. This study investigated whether recordings of sleep states in dairy cows also show a first-night effect. Non-invasive electrophysiological recordings were carried out on nine cows of the Swedish Red breed during three consecutive 24-hour periods (recording days 1–3). Overall, cows spent 12.9 ± 1.4 hours awake, 8.2 ± 1 hours ruminating, 57.2 ± 20.3 min drowsing, 44.1 ± 20.2 min in REM sleep and 64.3 ± 38.1 min in NREM (non-rapid eye movement) sleep (mean ± SD) and there were no significant differences between recording days in total duration for any of the sleep and awake states. However, the bouts of REM sleep and rumination were longer, and the awake bouts were shorter, at night time compared to daytime, regardless of recording day. The awake bouts also showed an interaction effect with longer bouts at daytime during day 1 compared to daytime on day 3. Data on sleep and awake states recorded in adult dairy cows during three consecutive 24-h periods showed great variation in sleep time between cows, but total time for each state was not significantly affected by recording day. Further and more detailed studies of how sleep architecture is affected by recording day is necessary to fully comprehend the first-night effect in dairy cows.

## Introduction

Modern research on sleep relies on non-invasive recordings of the electrophysiology of the brain, commonly using skin adhesive electrodes. Artefacts during the first night of electrophysiological recordings have been described in studies on humans and occur during sleep recordings in both the home environment and sleep laboratories [1, 2, 3]. Early work by Rechtschaffen and Verdone [1] showed that total sleep time did not differ between recording nights, but rapid eye movement (REM) sleep decreased and number of awakenings increased in human subjects during the first night compared with the fourth night of recording in a sleep laboratory. These findings were confirmed by Le Bon [3], who observed no difference in total sleep time, but reported that REM sleep did not return to a steady state until the fourth night of recording. These disturbances are known as the first-night effect, and are probably due to discomfort from the electrodes and the change of environment. As a consequence, first-night recordings are often discarded in human sleep studies [4, 5]. When estimating average total lying time in dairy cows on herd level, accuracy changed little between the 3- and 5-day sampling period [6].

Changes in environment have also been shown to affect behaviour and sleep distribution in cows. For example, moving cows between groups affect lying time and feeding behaviour on the day of regrouping but not subsequent days [7]. Sleep recordings have been carried out in adult dairy cows during 24-hour periods using permanently implanted brain electrodes [8]. The cows in Ruckebusch’s [8] study showed non-rapid eye movement (NREM) sleep for 3.25h, REM sleep for 45 min and they engaged in drowsing, described as an intermediate state with a reduced sensory threshold but no as low as for sleep, for 7.5h per 24h. Moreover they spent 12.5h awake, and it might be possible for some animals to tranced into NREM sleep during rumination [8]. According to that study and Ruckebusch [9], cows sleep both during the day and at night when on pasture, but only at night when indoors in a tie-stall environment. Ruckebusch [9] concluded that cows brought in from pasture to a tie-stall environment need 3-6 days to establish their indoor sleep pattern. However, if they were stanchioned in the tie-stall using a headlock gate, 14-21 days of adaptation were necessary to establish their indoor sleep pattern when brought in from pasture. In those early studies, it took longer for the cows to adapt to the recording equipment because of the more invasive method used for collecting electrophysiological data. This technique required the animals to be tethered or even stanchioned during recording. Thus, the lying behaviour and sleep pattern of the cows might have been disturbed. Whether the recording equipment and monitoring of the cows during the recording itself interfered with the sleep of the cows was not studied.

A non-invasive method for recording sleep, allowing the animals to move freely in a pen during recordings, has recently been developed for calves and adult cows [10, 11].

The aim of the present study was to investigate whether the non-invasive recording of sleep and awake states in dairy cows [11] cause “first-night effects” similar to what has been reported from sleep research in humans.

## Material and methods

Sleep recordings were conducted on nine cows of the Swedish Red breed during three consecutive 24-h periods, based on findings for variation in lying time by von Keyserlingk et al. [6], at the Swedish University of Agricultural Sciences, Uppsala, Sweden. Data recording was successful over these three days, but was interrupted for about 20 min each morning when data had to be transferred from the recording unit to the computer. The experimental design and handling of the animals during the experiment was approved by the Uppsala Local Ethics Committee (C313/10, 2010 Uppsala, Sweden). The recordings were conducted during winter, when the temperature in the experimental facility was 11 ± 2°C.

### Animals and housing

The experiment was designed to eliminate factors that could disturb the cows. Two days before the recording period, the cows were moved from a loose housing system to individual pens (3 m x 3 m) in a quiet room with no other cows except those participating in the study. The pens were equipped with headlocks to tether the cows at milking, a feeding trough for concentrate and silage, and a pressure valve water bowl. The cows had ad libitum access to water and silage during the recording period. Silage refusals were removed and fresh silage was given before morning milking, at lunchtime and before afternoon milking. Concentrate was provided to meet the individual requirements of each cow according to their milk production [12]. Bedding consisted of wood shavings and was replaced daily. Lighting was all artificial and light hours were 05.00-20.00 h, while at night a dim night-light was provided (approximately 30 lux). The cows were milked twice daily, at 06:00 h and 17:00 h. Average days in milk was 203 ± 20 and milk yield was 27 ± 4 litres per day. One cow was primiparous and the others were multiparous, in lactation number 2-4.

### Electrophysiological recordings

In the individual pens the cows were fitted with an udder holder and a textile halter. A minimum of 10 h before the recording started, they were shaved at the electrode attachment sites to ensure sufficient conduction.

**Fig 1:**
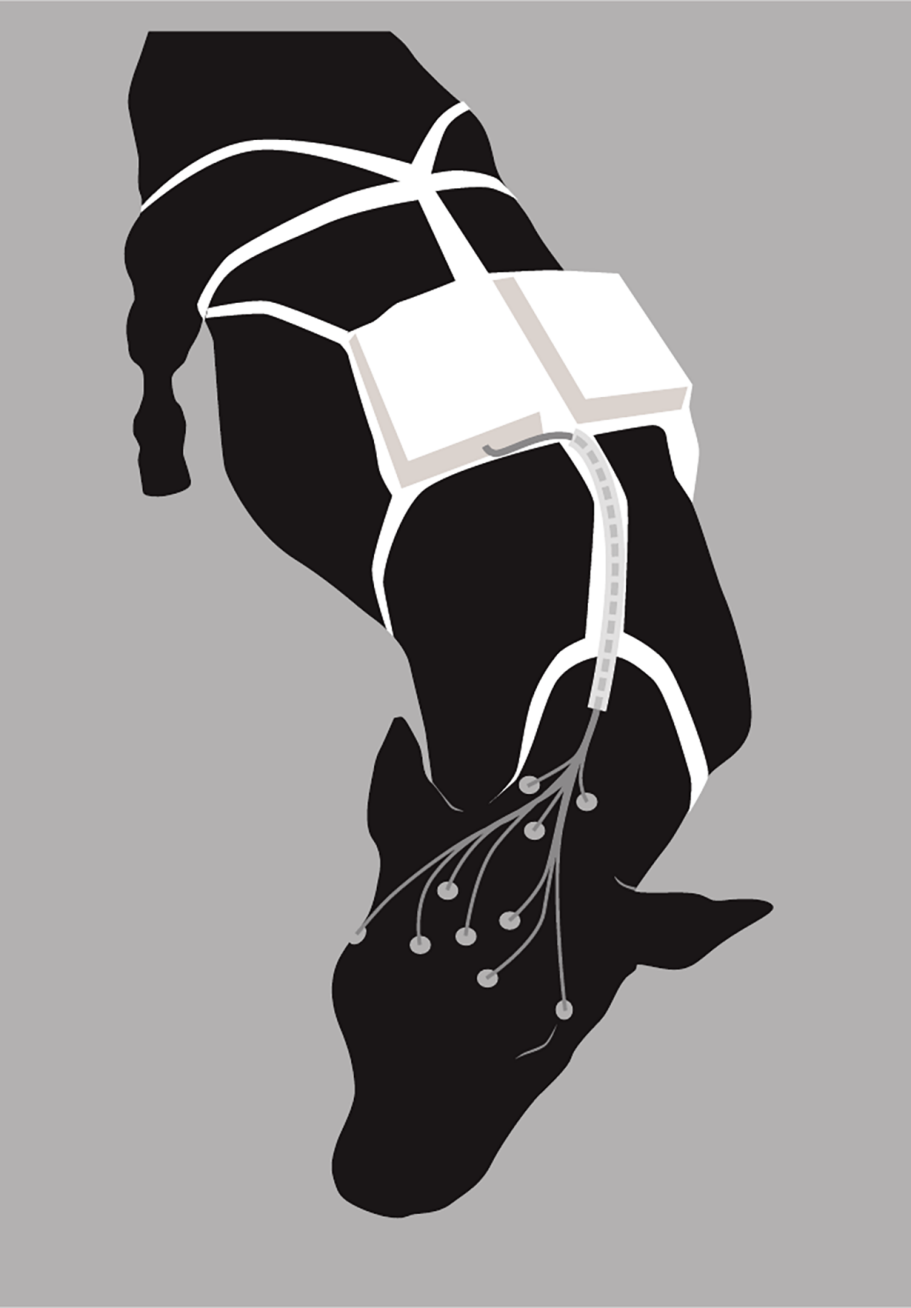
Electrode placement and equipment for non-invasive EEG recordings in dairy cows. The snap-on electrode wires were led through fabric tubes and connected to the recording device. A counter weight is attached on the back of the cow, opposite the device.

Electrodes (ø 3 cm) (Unilect, Unomedical Ltd, Birkerød, Denmark) were secured to the skin using tissue adhesive (VetBond™, 3M, St. Paul, MN, USA) and positioned as described by Ternman et al. [11] on the morning of recording day 1 (Fig 1). Four EEG electrodes were placed vertically two by two in lines drawn from the middle of the eye bulb up to the cranial part of the horn base in a square on the flat area of the cow’s forehead. The reference electrode was placed in the middle of the square and the ground electrode was placed caudally of the horn base. The electrodes were sometimes rubbed off against the interior by the cow, and was then rapidly replaced by new electrodes. Electrodes for measuring eye movements were positioned above and below one eye, and two electrodes for measuring neck muscle activity were fixed symmetrically on each side of the neck on cervical part of the trapezius muscle. All electrodes were connected to a mobile recording device (Embla Titanium, Embla Systems Inc., Broomfield, CO, USA) using snap-on cables (Embla Systems Inc., Broomfield, CO, USA). The cables, mobile recording device and a counter-weight were fixed to the udder holder on the back of the cow. Cables were bunched with plastic straps and fixed to the udder holder to reduce tension on the electrodes when the cow was moving and to reduce the risk of cables irritating the cow. The impedance of the electrode connections was checked and values between 0.2 and 5 kΩ were deemed acceptable. Two or three cows in neighbouring pens were recorded in parallel during each recording period.

Electrophysiological data were recorded by electroencephalography (EEG) for brain activity, electrooculography (EOG) for eye movements and electromyography (EMG) for muscle activity. The data were sampled at 256 Hz, as described by Ternman et al. [11]. The recording period started during morning milking at 06 h on day 1 and lasted for 72 h. During the whole 72-h recording period, the cows were closely monitored to ensure their well-being and to check that the electrodes were in place and that the equipment was working properly. The data were stored on the recording device until the end of each 24-h period and then downloaded onto a computer. This was done during morning milking. Data downloading took 20 min and a new recording was started immediately after the previous set of data had been downloaded.

### Analysis of electrophysiological data

The EEG, EOG and EMG data were analysed visually using digital sleep recording, monitoring and analysing software (RemLogic 2.0.1, Embla Systems, Broomfield, CO, USA) and scored in 30-s intervals for the vigilance states: awake, drowsing, NREM sleep and REM sleep [11]. Before scoring, the signals were filtered using the following individual thresholds: EEG 0.3–30 Hz; EMG >10 Hz and EOG 0.15–15 Hz. The signals were displayed and analysed in 30-second intervals and given a fixed scale; 50 μV for EEG and EMG and 500 μV for EOG. From each recording session, one good quality trace of the EEG and EMG, and both EOG traces were selected for visual analysis. Power spectrum was calculated using fast Fourier transform (FFT) with sample size of 2048 points and 50% overlap and was displayed in the frequencies 10–30 Hz and the power 0–1 μV^2^/Hz

The vigilance states (awake, NREM sleep and REM sleep) were scored according to the Ternman et al. [11]. REM sleep was scored when the EEG data displayed a desynchronized pattern with varying high and low amplitude, combined with very low muscle tone. NREM sleep was characterized by low frequency waves (amplitudes usually less than 15 μV) and was scored when the power spectrum showed less than 0.2 μV^2^/Hz in the higher frequencies (10–30 Hz) and EMG activity was reduced. Data was scored as drowsing when the EEG signal displayed a synchronized pattern and the activity in the higher frequencies (10–30 Hz) of the power spectrum was between 0.2 and 1.0 μV^2^/Hz, based on Ruckebusch’s [8] findings on cows, and with reference to Ternman et al. [11]. EMG signal during drowsing was reduced but could vary due to movements.

### Statistical analysis

Total time, number of bouts and bout duration per 24-h period were calculated for all scored vigilance states. In addition, these parameters were calculated for daytime (05.00-20.00 h) and night time (20.00-05.00 h) separately. A bout was defined as a period of a given vigilance state separated by at least one 30s epoch of a different vigilance state.

Differences between recording days (24-h periods) and time of the day were analysed using the PROC MIXED procedure in SAS (SAS 9.3, SAS Institute Inc., Cary, NC, USA). Recording day, time of the day and recording day × time of the day were included as fixed effects in the model and cow was included as a random effect. Values declared significant at *P* ≤ 0.05. Posthoc means separation for significant main effects was done using a T-test, data presented are LSM ± SE, if nothing else stated. Normality and equality of variance were checked by visual inspection of the residuals, and duration of awake bouts per recording day was log10 transformed due to non-normal distribution.

## Results

**Fig 2:**
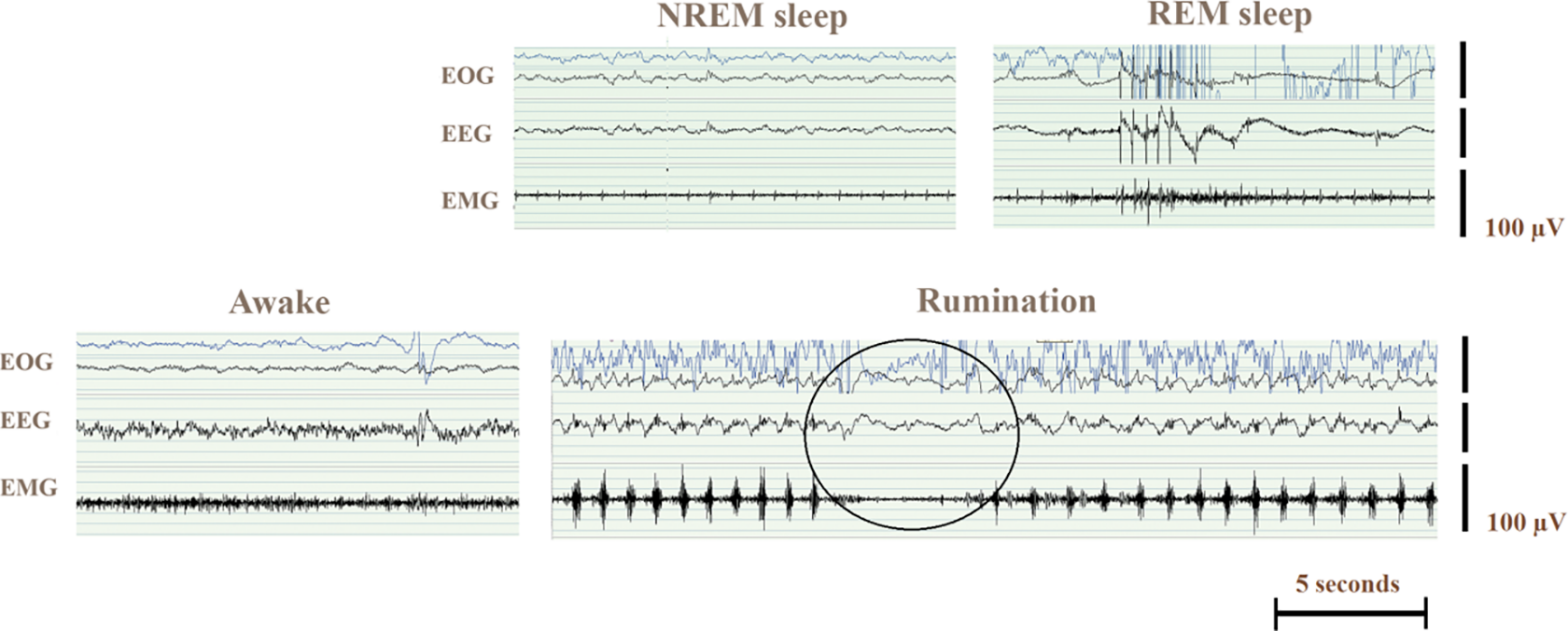
Electrophysiological recordings from one cow during non-rapid eye movement (NREM) sleep (15s), rapid eye movement (REM) sleep (15s), awake (15s) and rumination (30s). The artefacts in REM sleep data are the typical muscle twitches occurring during REM sleep. Eye movements recorded using electrooculography (EOG), brain activity using electroencephalography (EEG), and neck muscle tone using electromyography (EMG). Swallowing and eruption of a new bolus is encircled in the Rumination image.

Successful recordings were obtained during all 24-h periods. Typical electrophysiological curves for sleep and awake states and rumination are shown in Fig 2.

On average during the three consecutive recording days, cows spent 12.9 ± 1.4 hours awake, 8.2 ± 1 hours ruminating, 44.1 ± 20.2 min in REM sleep and 64.3 ± 38.1 min in NREM sleep (mean ± SD). Cows also spent 57.2 ± 20.3 min (mean ± SD) in the intermediate state drowsing. Overall, cows were awake more during daytime than at night (P < 0.001; Table 1); however, there were no effect of recording day or the interaction of recording day × time of the day. Cows showed more rumination, REM and NREM sleep at night as compared to daytime (P < 0.001; Table 1) but there were no differences between recording days or for the interaction of recording day × time of day.

**Table 1:** Percent of total duration (min) of vigilance states/day vs night split in Time of Day (TOD) and Recording Day (RecDay). Results shown as LSMeans ± SE.

		Time of day (TOD)		P-values			
Vigilance state	RecDay	Day	Night	RecDay × TOD	TOD	RecDay	DF
**NREM sleep % of total time** (min ± SEM in brackets)	1	2.5 ± 1.1 (21.0 ± 7.3)	6.0 ±1.1 (36.0 ± 7.3)	0.6060	< 0.001	0.1147	38
	2	3.4 ± 1.1 (28.8 ± 7.9)	8.4 ± 1.1 (49.3 ± 7.3)				
	3	2.8 ± 1.1 (24.8 ± 7.9)	6.6 ± 1.1 (37.5 ± 7.3)				
**REM sleep % of total time** (min ± SEM in brackets)	1	2.4 ± 0.7 (20.1 ± 4.9)	4.5 ± 0.7 (26.6 ± 4.6)	0.5235	< 0.001	0.1563	39
	2	2.6 ± 0.7 (22.2 ± 4.6)	5.0 ± 0.7 (29.3 ± 4.6)				
	3	2.1 ± 0.7 (18.3 ± 4.6)	3.1 ± 0.7 (18.1 ± 4.6)				
**Awake % of total time** (min ± SEM in brackets)	1	65.0 ± 2.5 (555.6 ± 9.5)	36.5 ± 2.5 (211.8 ± 9.5)	0.2037	< 0.001	0.1560	40
	2	62.9 ± 2.5 (532.6 ± 9.5)	37.4 ± 2.5 (215.7 ± 9.5)				
	3	64.4 ± 2.5 (564.4 ± 19.5)	43.9 ± 2.5 (245.6 ± 9.5)				
**Rumination % of total time** (min ± SEM in brackets)	1	27.5 ± 1.6 (233.5 ± 14.2)	47.6 ± 1.6 (277.2 ± 14.2)	0.1700	< 0.001	0.2379	40
	2	27.9± 1.6 (236.3 ± 14.2)	43.2 ± 1.6 (250.6 ± 14.2)				
	3	27.7 ± 1.6 (243.7 ± 14.2)	42.5 ± 1.6 (239.1 ± 14.2)				

Results shown as LSMeans ± SEM. P-values indicate significant differences for the interaction effect of Recording Day × Time of Day (RecDay×TOD), between day- and night time (TOD) and between recording days (RecDay). DF = degrees of freedom for each calculation.

Awake bouts were longer during daytime compared to night (P < 0.001; Table 2), and there was an interaction effect of recording day × time of the day with longer awake bouts during daytime on recording 1 compared to daytime on recording day 3 (11 min vs 9 min; P < 0.05). Both REM sleep bouts and rumination bouts were longer during night compared to daytime, but there were no differences between recording days and no interaction effect of recording day × time of day.

**Table 2:** Bout duration (min) of vigilance states split in Time of Day (TOD) and Recording Day (RecDay). Results shown as LSMeans ± SE.

		Time of the day		P-values			
Vigilance state	RecDay	Day	Night	RecDay × TOD	TOD	Recday	DF
**NREM sleep**	1	4.4 ± 0.6	4.0 ± 0.5	0.4322	0.6782	0.9967	388
	2	4.0 ± 0.6	4.4 ± 0.5				
	3	3.9 ± 0.6	4.4 ± 0.5				
**REM sleep**	1	4.0 ± 0.4	4.6 ± 0.4	0.3585	< 0.05	0.7913	254
	2	4.4 ± 0.4	4.6 ± 0.3				
	3	4.0 ± 0.4	5.2 ± 0.4				
**Awake Log10 (s),** (back-transformed in brackets, min)	1	6.5 ± 0.2 (11.5)	5.7 ± 0.1 (5.2)	< 0.05	< 0.001	0.4781	1011
	2	6.4 ± 0.1 (9.8)	5.6 ± 0.1 (4.7)				
	3	6.2 ± 0.1 (8.6)	6.0 ± 0.2 (6.5)				
**Rumination**	1	30.5 ± 2.0	32.4 ± 1.9	0.7850	< 0.05	0.2579	426
	2	26.3 ± 1.9	30.9 ± 1.9				
	3	29.3 ± 2.0	33.0 ± 2.1				

Results shown as LSMeans ± SEM. P-values indicate significant differences for the interaction effect of Recording Day and Time of Day (RecDay×TOD), between day- and night time (TOD) and between recording days (RecDay). DF = degrees of freedom for each calculation.

The percentage of NREM sleep bouts were higher at night compared to daytime (P < 0.001; Table 3), but there were no effect of time of the day or recording day. Percentage of REM sleep bouts did not differ between recording days or time of day, and did not show any interaction effect of recording day × time of day (Table 3). However, the percentage of awake and rumination bouts showed an interaction effect with more bouts occurring during daytime on recording day 3 compared to daytime on recording day 1 (P < 0.05 and 0.01, respectively), during night time on recording day 1 compared to night time on recording day 3 (P < 0.05 and 0.01, respectively), and daytime on recording day 3 compared to night time on recording day 3 (P < 0.05 and 0.01, respectively; Table 3). In addition, for rumination the percentage of bouts were higher also for night-time on day 1 compared to daytime on recording day 1 (P < 0.01; Table 3).

**Table 3:** Percentage of number of bouts per vigilance state/day vs night split in Time of Day (TOD) and Recording Day (RecDay). Results shown as LSMeans ± SE.

		Time of the day		P-values			
Vigilance state	RecDay	Day	Night	RecDay × TOD	TOD	Recday	DF
**NREM % of total number of bouts** (actual number in brackets)	1	35.8 ± 5.9 (5 ± 1)	64.2 ± 5.9 (9 ± 1)	0.6013	< 0.001	1.0000	38
	2	33.5 ± 5.9 (7 ± 1)	66.5 ± 5.9 (11 ± 1)				
	3	39.4 ± 5.9 (6 ± 1)	60.6 ± 5.9 (8 ± 1)				
**REM % of total number of bouts** (actual number in brackets)	1	44.9 ± 6.9 (5 ± 1)	55.1 ± 6.9 (6 ± 1)	0.1565	0.5669	1.0000	39
	2	44.0 ± 6.9 (5 ± 1)	56.0 ± 6.9 (6 ± 1)				
	3	56.2 ± 6.9 (5 ± 1)	43.8 ± 6.9 (3 ± 1)				
**AWAKE % of total number of bouts** (actual number in brackets)	1	45.5 ± 3.6 (15 ± 2)	54.5 ± 3.6 (18 ± 2)	< 0.05	0.8808	1.0000	40
	2	48.3 ± 3.6 (20 ± 2)	51.7 ± 3.6 (21 ± 2)				
	3	55.5 ± 3.6 (21± 2)	44.5 ± 3.6 (16 ± 2)				
**RUMINATION % of total number of bouts** (actual number in brackets)	1	46.6 ± 2.0 (7 ± 0.5)	53.4 ± 2.0 (8 ± 0.5)	< 0.01	0.4424	1.0000	40
	2	52.1 ± 2.0 (9 ± 0.5)	47.9 ± 2.0 (8 ± 0.5)				
	3	53.2 ± 2.0 (8 ± 0.5)	46.8 ± 2.0 (7 ± 0.5)				

Results shown as LSMeans ± SEM. P-values indicate significant differences for the interaction effect of Recording Day and Time of Day (RecDay×TOD), between day- and night time (TOD) and between recording days (RecDay). DF = degrees of freedom for each calculation.

## Discussion

This study examined whether total duration and the architecture for REM sleep and awake in dairy cows differ between recording days, or between the nights for each recording day respectively, using a non-invasive method recording brain and muscle activity and eye movements. We found that cows were awake more during daytime compared to night time and that each bout was longer during daytime compared to at night. This diurnal effect for awake is most likely due to the management routines as these are known to significantly affect dairy cow behaviour [13, 14]; in the present study all feeding and most cleaning of the pen occurred during daytime. Furthermore, the interaction between recording day and time of the day, with longer awake bouts during daytime on day 1 compared to day 3, could indicate that cows were affected by the equipment and/or housing situation. The recordings were always started during morning milking on recording day 1, and cows would potentially have been more affected by the equipment during daytime on day 1. The first-night effect in human subjects includes a prolonged latency to fall asleep [4, 2] and an increased number of awakenings on the first night compared to night 2-4 [1]. However, in our study there was no effect on number of awake bouts between the recording days, suggesting that the character of a possible first-night effect in cows might differ from what has been shown in human subjects.

Alteration of REM sleep time and architecture is an additional aspect of the first-night effect in human subjects [15, 3]. Cows in the present study displayed several REM sleep bouts per recording day indicating a polyphasic sleep pattern. This type of sleep architecture is common for most mammals and has also been shown in sleep studies on dairy cows [8] and dairy calves [10]. The only effect on REM sleep in the present study was the time of the day, with longer REM sleep bouts during night time compared to daytime.

Rumination was scored as a distinct vigilance state, since electrophysiological data during rumination are dominated by artefacts from the chewing [11]. Although never fully examined, it was proposed by Bell and Itabishi [16] and by Ruckebusch [8] that ruminants can engage in the vigilance states awake and NREM sleep but not REM sleep [17] during rumination. Similarly to Ruckebusch [8], we found that cows spent about 8 hours per day ruminating and there were no differences between recording days. As for the REM sleep bout lengths, rumination bouts were longer during night-time compared to daytime, but there was nothing that could indicate a “first-night effect”, such as effect of recording day or an interaction between recording day × time of day. However, it might suggest that also rumination bouts are affected by management routines, such as feeding, milking and cleaning, and that cows engage in longer bouts of behaviours preferably performed while lying down when there is little activity in the barn. The decrease in number of rumination bouts on daytime during recording day 1 compared to daytime during recording day 2 might indicate that cows were not comfortable during the first day of recording as compared to the second day of recording. However, the total time cows spent ruminating showed no interaction effect for recording day and time of day and neither did length of each bout, why this is believed to be of less importance.

Total NREM sleep duration per day in the present study was significantly shorter, just over an hour, than what has been presented by Ruckebusch [8], about 3 hours. In the latter study, stage of lactation, pregnancy status and age are not reported, but these factors have been shown to alter sleep time in other species [18, 19]. Ruckebusch [8] also reported that cows frequently drowse during rumination and that cows may fall asleep for short periods during rumination. Using the non-invasive recording technique, vigilance state during rumination cannot be estimated due to the muscle artefacts from chewing, thus we might have underestimated the amount of NREM sleep the cows had in our study. Cows showed more NREM sleep at night compared to daytime, which confirms findings by Ruckebusch [8]. Ruckebusch [9] concluded that when cows are housed indoors their sleep is restricted to night time, this was however not the case in the present study. As mentioned previously, it is possible that the cows studied by Ruckebusch [8, 9, 17] preferred to rest and sleep after staff working hours, when there was less disturbance in their immediate environment.

The variation between cows of total duration, bout lengths and number of bouts in awake and sleep states was large making it difficult to establish a pattern for the first-night effect with this limited sample size. However, there seem to be a within cow consistency indicating that using the same individuals to register sleep under different conditions, like stage of lactation or housing system, is more important than the number of days recorded. More studies with a larger number of animals are needed to establish the detailed first-night effects of sleep recordings in dairy cows, but for total amount of sleep and awake states there seem to be no difference between recording days. This is in agreement with studies in human subjects where total sleep time also was unaffected by day of recording [2, 3]. Type of recording method should be taken into account as it may affect the total duration and the architecture of sleep since cows may be more or less disturbed by the equipment. A minimally invasive method with wireless data transfer for the polygraphic data would perhaps disturb the cows less.

## Conclusion

Data on sleep and awake states recorded in adult dairy cows using a non-invasive electrophysiological method applied during three consecutive 24-h periods showed great variation in sleep time between cows, but the variation between days in total time for any of the recorded sleep and awake states was not as pronounced. This indicates that events recorded during the first 24-h period can be regarded as representative if the total amount rather than the architecture of the sleep and awake states are of primary interest. Further and more detailed studies, with a larger sample size, of how sleep architecture is affected by recording day is necessary to fully comprehend the first-night effect in dairy cows.

## Supporting information

S1 Table Raw data from the study. Duration is shown in seconds, abbreviations for events are W = awake, N1 = drowsing, N2 = NREM sleep, N3 = REM sleep and R = Rumination, and RecDay corresponds to recording day.
(XLSX)
